# Changes in serum concentration of perioperative inflammatory cytokines following the timing of surgery among mild–moderate traumatic brain injury patients and factors associated

**DOI:** 10.3389/fneur.2024.1484742

**Published:** 2024-12-18

**Authors:** Hervé Monka Lekuya, Stephen Cose, Marjorie Nakibuule, Gift Ahimbisibwe, Anthony Fuller, Larrey Kasereka Kamabu, Emmanuel Biryabarema, Geoffrey Olweny, David Patrick Kateete, Anthony Kirabira, Fredrick Makumbi, Jelle Vandersteene, Edward Baert, Moses Galukande, Jean-Pierre Okito Kalala

**Affiliations:** ^**1**^Department of Surgery, College of Health Sciences, Makerere University, Kampala, Uganda; ^2^Department of Human Repair, Neurosurgery, Ghent University, Ghent, Belgium; ^3^Medical Research Council, London School of Hygiene and Tropical Medicine, Immunology, Entebbe, Uganda; ^4^Duke Global Neurology and Neurosurgery, Duke University, Durham, NC, United States; ^5^Department of Molecular Biology, College of Health Sciences, Makerere University, Kampala, Uganda; ^6^School of Public Health, College of Health Sciences, Makerere University, Kampala, Uganda

**Keywords:** neuro-inflammation, perioperative serum levels inflammatory cytokines, systemic inflammatory response, timing of surgery, traumatic brain injury

## Abstract

**Background:**

The safe timing window for surgery during the acute phase of inflammation due to traumatic brain injury (TBI) has not been studied extensively. We aimed to elucidate the relationship between the timing of surgery and changes in perioperative serum levels of inflammatory cytokines and factors associated to optimize TBI management in low-middle-income countries.

**Methods:**

A prospective cohort study was conducted among TBI Patients with depressed skull fractures with a GCS > 8 operated at different timing from injury and followed up peri-operatively. We collected the clinical-radiological data, as well as pre-and postoperative venous samples from participants; we then did Luminex Assay to quantify the serum levels of pro/anti-inflammatory cytokines using the kits of 96-well human cytokine “27-Plex-Assay (#M500KCAF0Y®).” We performed the analysis with STATA version 17 and R_studio applying both descriptive and inferential methods.

**Results:**

We enrolled 82 TBI patients with a median (IQR) age of 25.5 (20–34) years, and the majority were male (85.4%). There were 48.8% victims of assaults, and 73.2% had a post-resuscitation admission GCS of 14–15. There were 38 (46.3%) who were operated within 48 h of injury versus 44 (53.7%) after 48 h. Serum levels of TNF-*α* were significantly higher after surgeries done >48 h compared to those done ≤48 h (*p* = 0.0327); whereas, the difference in post-operative mean serum levels of IL-10 was significantly increased in patients who developed later SSI compared to those who did not (11.56 versus −0.58 pg./mL, *p* = 0.0489). In multivariate analysis, the history of post-traumatic seizure (PTS) was associated with a postoperative increase in TNF-*α* (*p* = 0.01), the hemoglobin of 10–12 with a postoperative decrease of IL-4 (*p* = 0.05); the presence of focal neurological deficit was associated with a significant postoperative increased of TNF-α, IL-6, and IL-4 (*p* = 0.05). The presence of extra-axial hemorrhage was associated with a postoperative increase of IL-10 (*p* = 0.05).

**Conclusion:**

Delayed surgical intervention beyond 48 h post-injury in mild–moderate TBI patients results in a significantly increased postoperative inflammatory response, as evidenced by elevated serum levels of TNF-*α* and IL-6. Neurological deficits, PTS, reduced hemoglobin rate, and extra-axial intracranial hemorrhage are factors associated with this heightened response.

## 1 Introduction

Traumatic brain injury (TBI) is one of the leading causes of worldwide mortality, and disability (1, 2). After surviving the initial death from TBI, subsequent morbidity and mortality due to TBI are attributed to the systemic inflammatory response (SIR) through neuro-inflammation (3–6). SIR is the immune system’s response to harmful stimuli, such as a large number of damaged cells from trauma injury, pathogens, toxic compounds, or irradiation, and acts by removing harmful stimuli and initiating the healing process (7–9). SIR is orchestrated by the release of inflammatory cytokines, which are a broad class of small, secreted proteins that act locally or systemically to trigger activities among immune cells; (10) thus, they play a key role in the pathophysiology of TBI-induced neuro-inflammation. The brain is considered an ‘immune-privileged’ organ that is not largely affected by the SIR until the natural brain–blood-barrier (BBB) is breached as is the case in TBI (11). Indeed, following the primary insult to the brain during the TBI process, a systemic stress response is triggered; peripheral immune cells invade the BBB, and the residual microglia and macrophages may be activated locally (11). This focal neuro-inflammation process will not be restricted to the affected region in the brain, but progressively will disseminate beyond (12); there will be a coordinated activation of signaling pathways that regulate inflammatory mediator levels in resident tissue cells and recruited inflammatory cells, and then a release of pro-and anti-inflammatory cytokines into the systemic circulation within minutes to hours; this will cause important biological and sub-clinical changes from neuro-inflammation to a certain extend of brain edema, exacerbate neuronal dysfunction within hours to days (3–6, 9, 13). At the initial stage of the acute phase response, SIR is normally activated without being very harmful, since the cytokines’ production will be normalized later if its amplitude stays within the homeostatic range (11). However, when the pro-inflammatory cytokines, such as tumor necrosis factor-alpha (TNF-*α*), interleukin 6 (IL-6), and many others are released excessively, their action becomes destructive (14), and will be associated with poor outcomes through organ dysfunction and altered metabolism (5, 15). Anti-inflammatory cytokines, such as interleukin 10 (IL-10) and many others, may play a neuroprotective role in down-regulating the inflammatory response and promoting possible healing to homeostasis (16). The second insult to the brain following TBI is due to SIR induced by the ongoing ischemia and increased intracranial pressure (ICP), major surgery under general anesthesia, or other systemic causes that can further induce a prolonged deleterious immune response on top of the homeostatic responses from the acute phase of the injury; this will worsen the neurological outcome through an excessive neuro-inflammation with dysfunction, and a variety of complex homeostatic responses to trauma in regards to body volume loss, under-perfusion, starvation, tissue damage, and ongoing oxidative stress (5, 6, 17–22). Surgery is often one of the primary management strategies in TBI by repairing traumatic lesions and relieving ICP (23, 24). In low and middle-income countries (LMIC), there has been observed in the last decade an increase in TBI incidence due to road traffic crashes (RTC) and assaults, with subsequent increased number of TBI surgeries (25, 26). Indeed, up to 22% of moderate to severe TBI patients undergo major TBI surgery in LMIC (26), and while the most performed TBI surgery is the elevation of depressed skull fracture (DSF) (27). Major surgery under general anesthesia, well-known additional inflammatory stimuli, leads to the so-called “surgery-induced stress,” an adaptive physiological response of the body in the context of an ongoing SIR, especially by receiving general anesthetic agents, intravenous fluids, additional surgical incision, tissue dissection, and intraoperative blood loss (21, 28–30). In the perioperative period, there is activation of catabolism due to inadequate nutrition with additional release of inflammatory cytokines and other immunological mediators (28). Thus, the surgical timing regarding the neuro-inflammatory process during its acute phase may have a relationship with the dynamic of inflammatory cytokines production. Regarding the surgical timing in TBI, according to the recent guidelines of the Brain Trauma Foundation (23), there is still a lack of evidence-based recommendations of the optimal timing of TBI surgery, especially for relatively stable TBI patients as is the case for patients with DSF. This leads to a hypothetical conflicting choice of performing an early versus delayed TBI surgery based on the hospital duration of stay rather than the actual surgical outcomes. Recent studies done in Uganda observed a longer delay in surgical timing of mild–moderate TBI when comparing severe TBI (31). Previous studies recommended earlier interventions of TBI whenever indicated to reduce the incidence of surgical site infections (SSI), but still, the cutoff timing was not clearly suggested (32, 33). With the advent of the damage control approach, emphasis is made on dealing first with the resuscitation of life-threatening situations related to the lethal triad during the acute phase of trauma and taking patients with major trauma into the operating theater to perform preferentially life-saving surgical procedures in the early hours of the day one of injury (34, 35). Thus, the question of performing a major surgery during the early hours of the acute phase of the inflammatory response of trauma stress became controversial for stable TBI patients. Indeed, the trend of inflammatory cytokines after trauma stress during the perioperative period is yet to be investigated; the optimal surgical timing window has not been studied in TBI, and most studies recommend an earlier intervention without clearly defining the optimal bracket of a safe surgical timing from the injury if indicated. We hypothesized that the timing of major TBI surgery under general anesthesia in the acute phase of injury may modify the expression of perioperative inflammatory cytokines that could be clinically relevant for the surgical outcomes. We aimed to elucidate the relationship between the timing of surgery, changes in serum concentration of perioperative inflammatory cytokines, and associated factors to define a safe window of surgical timing and optimize TBI management in LMIC.

## 2 Methods

### 2.1 Study design, setting, and participants

This was a prospective cohort study among TBI patients exclusively with DSF admitted in the Accident and Emergency Department (A&E) of MNRH, Kampala, Uganda as part of the DESTINE Study project between March 2021 to February 2022. The MNRH is the largest public tertiary hospital in Uganda and also the teaching hospital of Makerere University College of Health Sciences. Its A&E serves as an equivalent of a “level one trauma center facility” in Uganda and treats a high volume of trauma patients referred from all over the country. The study participants were TBI patients of all ages with DSF evidenced on a brain CT scan exclusively, admitted in the A&E or at the referring hospital within 6 h of injury, with a post-resuscitation GCS above 8, with SpO2 > 94% in room air, hemodynamically stable, with a hemoglobin level above 9 g/dL, who required an operation of elevation of DSF as a primary indication, and whose informed written consent was obtained.

We excluded patients with a history of blood transfusion before the recruitment, hemodynamic instability, with a penetrating mechanism, evidence of scalp infection, gross wound contamination with or without exposed bone fragments/fungi cerebri, multiple (more than two) scalp wounds or skin loss, with signs of infections/inflammatory conditions before surgery, with a DSF over the posterior two-thirds of the superior sagittal sinus, patients re-admitted after a non-surgical management attempt, and patients with history of head trauma, brain surgery, steroids treatment, or with comorbidities.

### 2.2 Sampling and study variables

Patients were recruited consecutively until the targeted sample size of 82 patients was attained, nested from the DESTINE study. The main exposure variable was the timing of surgery which was defined as early surgical intervention when done within 48 h after injury, and delayed surgical intervention when done beyond 48 h following injury. This surgical timing cut-off of “within 48 h” (early surgery) versus “after 48 h” (delayed surgery) was chosen initially based on the cut-off of early versus delayed surgical intervention regarding the post-traumatic inflammatory process in poly-trauma patients (35), and posterior in combination with the observed plotted curves of daily trends of pre-and postoperative concentration of cytokines of TBI patients from this current study. Other independent variables included the socio-demographic characteristics (age and sex), mechanisms of injury, association of long-bone fractures, post-resuscitation GCS score, neurological deficit, post-traumatic seizure (immediate, early, and late), pre-operative hemoglobin rate, type of DSF (simple or compound), location (frontal, parietal, temporal, or occipital) of DSF, head CT scan findings (underlying cerebral contusion, extra-axial intracranial hemorrhage, status of the basal cisterns, presence of midline shift), ASA classification, duration of anesthesia, duration of surgery, and dural tear findings. The main dependent variables were changes in pre-and postoperative serum levels of the known pro- (IL-6, IL-1b, TNF-*α*, & IL-8) and anti-inflammatory (IL-4, IL-10, & IL-13) cytokines. The secondary dependent variables were the occurrence of SSI within 3 months which was measured as a dichotomous outcome (SSI versus no SSI), the postoperative hospital length of stay (in days), 3 months hospital outcomes after surgery, and overall extended Glasgow Outcome Scale (eGOS) at 6 months after the initial surgery. In this study, we operationalized that the initial inflammatory response is mainly induced by trauma (supported by our narrow selection criteria), and the secondary inflammatory response was induced or modified by surgery and anesthesia.

### 2.3 Data collection and research procedures

#### 2.3.1 Recruitment, initial pre-operative clinical assessment, and management

All TBI patients with suspicious DSF underwent initial care according to the Advanced Trauma Life Support (ATLS) protocol. They continued receiving routine trauma care such as analgesia, tetanus toxoid injection, soft tissue suturing debridement, and so on. After obtaining a brain CT scan, patients with a confirmed diagnosis of DSF were identified by an active screening research team and were approached (or their next-of-kin) for consent and recruitment into the study. Once recruited, patients were thereafter tracked by using a color-coded dot sticker on a paper-based file until the time of the surgery of the DSF. The timing of surgery for patients was not decided by the investigators, but by the neurosurgery team on duty based on their readiness for patients’ investigations or the availability of theater space that is frequently given to emergency life-threatening cases. The timing of surgery was recorded as the duration between the estimated time of injury and the surgical incision after general anesthesia. In the preoperative period, a few minutes before induction of general anesthesia, a peripheral venous blood sample was taken, and thereafter patients had TBI surgery for elevation of the DSF as a standard practice in neurosurgery. In the early postoperative period within the next 6 to 10 h from the time of recovery from anesthesia (when the patient is fully awake, or at least at the initial pre-operative GCS), again another similar blood sample was taken. Indeed, some adult patients had randomly additional pre- or post-operative samples from the time of consent to discharge.

#### 2.3.2 Plasma separation

Each peripheral venous whole-blood sample of about 4 mL for adults and 2 mL for children of less than 8 years was withdrawn aseptically, collected in a green top vacutainer (Lithium heparin), put on a safety bag, subjected to a strict protocol of sample preservation, and sent to the nearby immunology laboratory of Makerere University for plasma separation. Samples were centrifuged at 18° Celcius (room temperature) for 10 min at 1200 rpm, the supernatant was removed to clean tubes, and the samples were further clarified by centrifugation at 18° Celcius for 10 min, 10,000 X g. The resulting platelet-depleted plasma fraction was divided into aliquots, transferred into cryotubes, and stored at −80° Celsius for several months until the assay was done all at once.

#### 2.3.3 Patients’ postoperative management and follow-up

Patients were followed up in the neurosurgical intensive care (ICU)/high-dependency Unit (HDU), or wards during the postoperative period up to discharge. Routine postoperative care was given to patients as requested in the postoperative instructions (analgesia, antibiotics, anti-epileptics drugs, physiotherapy, etc.). Any occurrence of seizures, wound infection, other types of morbidity, mortality, and the duration of the hospital stay was recorded from the enrollment into the study up to discharge, as well as in the outpatient clinics during suture removal, at 1 month, at 3 months, and 6 months by phone call appointment with random physical review whenever relevant for clinical inspection (wound inspection, major complaints, etc.), or during the regular hospital appointment for the neurosurgery outpatient clinics’ review.

#### 2.3.4 Multiplex Luminex assay of cytokines

All supernatants were thereafter retrieved from the storage lab for Luminex Assay of serum levels of pro- (IL-6, IL-1β, TNF-*α*, & IL-8), anti-inflammatory cytokines (IL-4, IL-10, & IL-13), and other additional types cytokines (IFN-*γ*, IL-1ra, IL-2, IL-5, IL-7, IL-9, IL-12, IL-15, IL-17), chemokines(Eotaxin, IP-10, MCP-1, MIP1a, MIP1b, RANTES), and growth factors(bFGF, GCSF, GMCSF, PDGFbb, VEGF) available in the kit. The samples’ assay was done on the same day. The 96-well Bio-Plex Pro™ Human Cytokine “27-Plex Panel (catalog #M500KCAF0Y®, Bio-Rad, Hercules, CA, United States)” was used. Serial dilution techniques were used to quantify the concentrations of cytokines, following the manufacturers’ recommendations, using Bio-Plex 200 System (Bio-Rad). The kits’instruction manual and procedures are available at this link: https://www.bio-rad.com/sites/default/files/webroot/web/pdf/lsr/literature/10000076326.pdf. Samples below the limit of detection were assigned values corresponding to half of the lowest standard value and those above the highest limit of detection were given the value of the highest standard.

### 2.4 Data management and analysis

Raw data were entered into an Excel spreadsheet, cleaned, and exported to STATA version 17 (StataCorp, College Station, TX, United States) and in R studio for analysis using both descriptive and inferential methods.

For baseline characteristics, numerical data were summarized using mean and standard deviation (SD) if normally distributed, and median and interquartile range (IQR) if skewed, whereas categorical data were summarized as frequencies and percentages. The categorical baseline variables were compared between the early and delayed surgical groups using the Chi-square, whereas the Mann–Whitney U tests were used for continuous baseline variables.

The relationship between the standardized concentrations of the 27 cytokines and the duration (in days) between injury and sample collection was assessed using a matrix plot. The plots for the pre-and postoperative serum cytokines were further stratified by the nature of inflammatory actions of the cytokines (pro- and anti-inflammatory). We also visually compared the levels of inflammatory cytokines between the delayed and early groups using the box plots. Pre-and postoperative levels of inflammatory cytokines and their change in concentration were compared between the categories of surgical timing, neurological variables, radiological variables, and clinical outcomes variables, using the t-tests and analysis of variance as appropriate. Statistical significance was set at *p* < 0.05.

The factors associated with the changes in the levels of cytokines (mainly IL-4, IL-6, IL-10, and TNF-*α*) were determined using the linear regression models. These four particular cytokines were considered by the fact that they were the most reported in the literature among the pro- and anti-inflammatory cytokines in TBI studies (36–39). The concentrations of cytokines were Log-transformed to meet the assumption of the linear regression model. Bivariate analysis was conducted and variables that yielded a *p*-value <0.25 were considered for multivariate analysis. In multivariate analysis, the model was logically developed in a stepwise approach; variables such as age and sex were considered regardless of their significance in the models. Variables that yielded a p-value of <0.05 were considered significant at a 95% confidence interval (95%CI). The results were reported at adjusted coefficient (aCoef.).

### 2.5 Ethical considerations

This study is a subset of the research project on the surgical timing of TBI patients in Uganda (DESTINE-study) that obtained ethical approvals at all levels from the Makerere University School of Medicine Research and Ethical Committee, from the MNRH as an administrative hospital clearance, and from the national ethical council of Uganda registered as HS1284ES. Patients or the legal next-of-kin consented before recruitment.

## 3 Results

**Figure 1:**
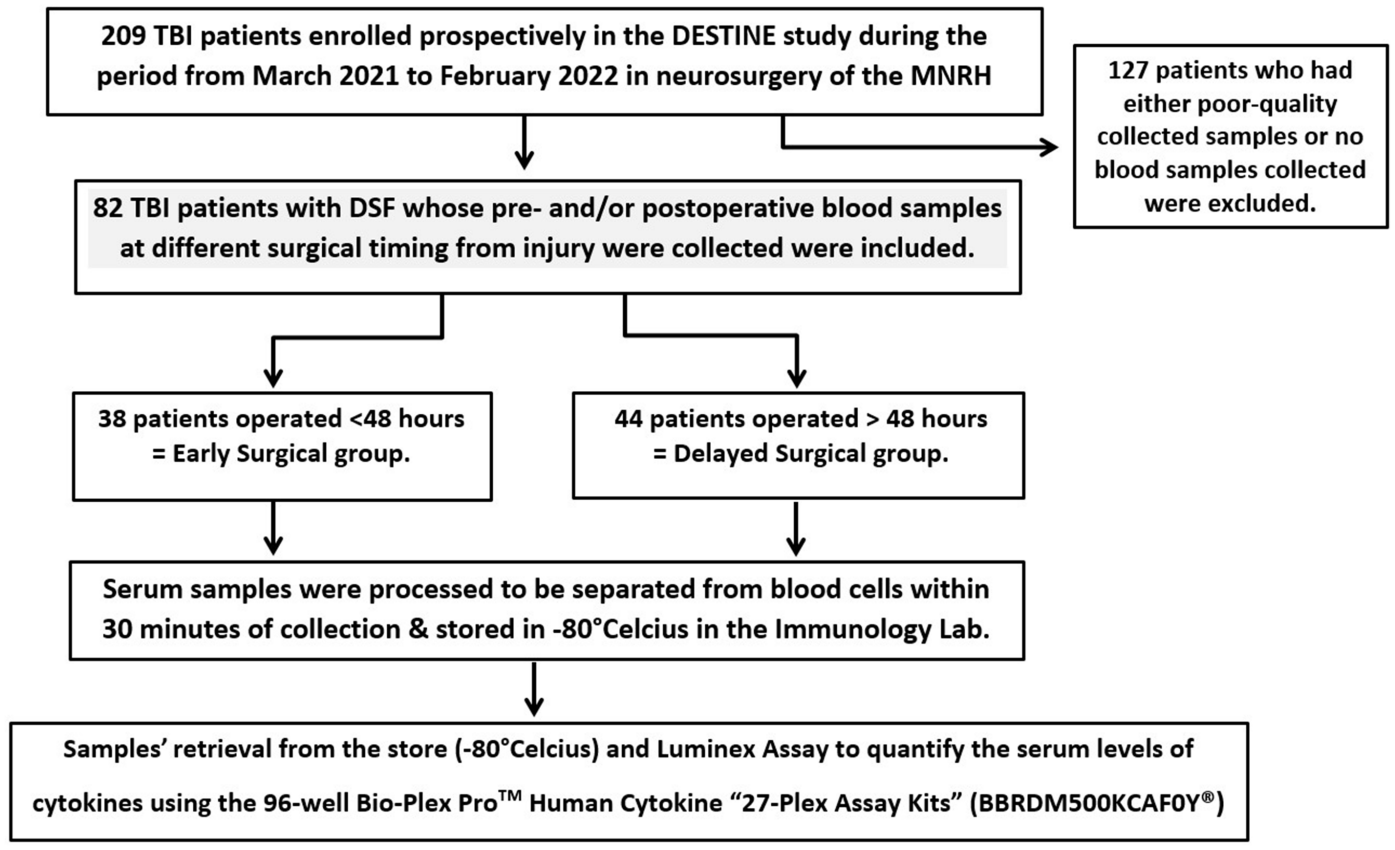
Patients and samples processing flow chart.

We included a total of 82 operated patients with DSF in this study based on the study criteria with pre- and postoperative samples taken during the acute phase of injury as shown in the patients’ flowchart (Figure 1). There were 38 (46.3%) who were operated within 48 h of injury (early group) and 44 (53.7%) after 48 h (delayed group).

### 3.1 Demographics, injury factors, clinical presentations, and neuro-imaging findings

The majority of them were male (70/82 = 85.4%) and the median age was 25.5 years (IQR = 20–34). There were 48.8% victims of assaults and 28.1% were injured by road traffic crashes (RTC). Other mechanisms were 23.2%. There were 73.2% of patients who had a post-resuscitation admission GCS of 14 and 15. There were 25.6% of patients who presented with a focal neurologic deficit at admission (hemiparesis, monoparesis, and/or cranial nerve palsy). There were 37.8% of patients who had a history of immediate or early post-traumatic seizures (PTS) almost as generalized tonic–clonic convulsion. There were 7.3% of patients who presented with associated long bone fractures. All patients had at least one brain CT scan following admission. The parietal and frontal bones were the most frequently involved (48.6 and 47.6% respectively). The brain CT scan shows frequently underlying cerebral contusion (84.2%), underlying extra-axial hemorrhage (29.3%), basal cisterns open (52.4%), and midline shift <5 mm (8.5%) as shown in Table 1. The mean pre-operative hemoglobin rate was 12.2 (± 2.3) g/dL before surgery, and the pre-operative ASA class 2 was the most common type (78.1%; Table 1).

**Table 1:** The baseline demographic, clinical, and radiological characteristics of the participants.

Variable	Overall	Surgical intervention timing group		Fishers exact
	*N* (%), 82	Early (≤48 h) *N* (%); 38(46.3%)	Delayed (>48 h) *N* (%); 44 (53.7%)	*p*- value
Age (Years), median (IQR); Mann-W. test	25.5 (20–34)	26 (18–36)	25 (20–34)	0.2299
Sex of the patients				
Female	12 (14.6%)	3 (25.0%)	9 (75.0%)	0.1086
Male	70 (85.4%)	35 (50.0%)	35 (50.0%)	
Mechanism of injury				
Assault	40 (48.8%)	18 (45.0%)	22 (55.0%)	0.9717
Passenger motor cycle RTC	23 (28.1%)	11 (47.8%)	12 (52.2%)	
Others	19 (23.2%)	9 (47.4%)	10 (52.6%)	
Post-resuscitation admission GCS				
9–13	22 (26.8%)	11 (50.0%)	11 (50.0%)	0.6875
14–15	60 (73.2%)	27 (45.0%)	33 (55.0%)	`
Post-traumatic seizures (Immediate or early)				
No	61 (62.2%)	28 (45.9%)	33 (54.1%)	0.8917
Yes	21 (37.8%)	10 (47.6%)	11 (52.4%)	
Associated long bone fractures				
No	76 (92.7%)	34 (44.7%)	42 (55.3%)	0.2997
Yes	6 (7.3%)	4 (66.7%)	2 (33.3%)	
Neurological focal deficit				
No deficit	61 (74.4%)	28 (34.1%)	33 (40.2%)	0.8917
Deficit	21 (25.6%)	10 (12.2%)	11 (13.4%)	
Type of DSF				
Simple	42 (51.2%)	22 (55.0%)	18 (45.0%)	0.12495
Compound	40 (48.8%)	16 (38.1%)	26 (61.9%)	
Location of DSF				
Frontal	34 (41.5%)	18 (52.9%)	16 (47.1%)	0.2574
Parietal	24 (29.3%)	13 (54.2%)	11 (45.8%)	
Temporal	14 (17.1%)	4 (28.6%)	10 (71.4%)	
Combined location	10 (12.2%)	3 (30.0%)	7 (70.0%)	
Presence of contusion in brain CT scan				
No	13 (15.9%)	5 (38.5%)	8 (61.5%)	0.5345
Yes	69 (84.2%)	33 (47.8%)	36 (52.2%)	
Extra-axial hemorrhage in brain CT scan				
No	58 (70.7%)	28 (48.3%)	30 (51.7%)	0.5850
Yes	24 (29.3%)	10 (41.7%)	14 (58.3%)	
Status of basal cisterns in brain CT scan				
Absent	11 (13.4%)	3 (27.3%)	8 (72.7%)	0.3332
Compressed	28 (34.2%)	15 (53.6%)	13 (46.4%)	
Open	43 (52.4%)	20 (46.5%)	23 (53.5%)	
Presence of midline shift ≥ 5 mm in brain CT				
No	75 (91.5%)	36 (48.0%)	39 (52.0%)	0.3242
Yes	7 (8.5%)	2 (28.6%)	5 (71.4%)	
Pre-oper. Hemoglobin (g/dL), mean (SD)	12.2 (±2.3)	12.7 (±2.2)	11.8 (±2.3)	0.0975
ASA classification				
Class I (relatively normal health)	5 (6.1%)	1 (20.0%)	4 (80.0%)	0.4704
Class II (mild systemic disease)	64 (78.1%)	31 (48.4%)	33 (51.6%)	
Class III (severe systemic disease)	13 (15.8%)	6 (46.2%)	7 (53.8%)	
Intraoperative finding of a dural tear				
No	43 (52.4%)	23 (53.5%)	20 (46.5%)	0.1729
Yes	39 (47.6%)	15 (38.5%)	24 (61.5%)	
Duration of surgery (min), mean (SD)	82.3 (±35.8)	74.8 (±22.8)	88.8 (±43.4)	0.0791
Surgical Site infection				
No	66 (80.49%)	34 (51.5%)	32 (48.5%)	0.0563
Yes	16 (19.51%)	4 (25.0%)	12 (75.0%)	

IQR, Interquartile range; SD, Standard deviation.

### 3.2 Management and surgical outcomes

Patients received routine trauma care from admission (100%), analgesics (100%), prophylactic anti-epileptic drugs (100%), tranexamic acid (69.5%), pre-operative osmotherapy (43.9%), and prophylactic intraoperative antibiotics (100%). There were 51% clinically classified as simple versus 49% as compound DSF. The mean duration of the general anesthesia was 122.3 (SD: ± 42.1) minutes, and of the surgical procedure was 82.3 (SD: ±35.8) minutes.

Surgical management consisted of debridement and elevation through craniotomy (100%) and additional hematoma evacuation. About 47.6% of patients had an intraoperative finding of dural tear, and subsequent duroplasty was performed. Patients had routine post-operative care in the HDU/ICU and ward for 24 h, clinical reviews, and wound care. There were 16 (19.51%) who developed SSI (Table 1). Most patients had a good recovery or improvement rate (65/82, 79.3%) after the initial surgery of DSF within 3 months. Two patients died in the features of intracranial infection. The overall mean length of stay in the hospital was 7.85 days (SD ± 4.84).

### 3.3 Serum levels of cytokines, chemokines, and growth factors

**Figure 2:**
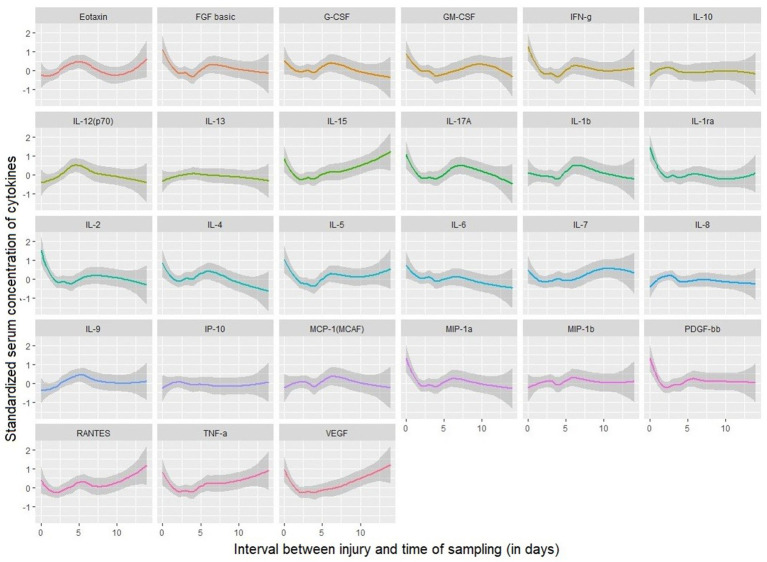
Standardized pre- and postoperative serum levels of 27 cytokines over timing between injury and sampling collection.

Standardized serum mean concentrations from pre- and postoperative samples were plotted in Figure 2; on data visualization, the transition from day-two to day-three of injury constitutes the most frequent time-point of the shifting of concentrations in the curves in either direction (increased or decrease). Regarding the inflammatory cytokines of interest in this study, serum levels of the pro- (IL-6, IL-1b, TNF-*α*, and IL-8) and the anti-inflammatory cytokines (IL-4, IL-10, & IL-13) of TBI patients measured pre- and postoperatively based on independent neurological and neuro-radiological variables are shown in Tables 2–5.

**Table 2:** Means serum levels of inflammatory cytokines according to surgical timing group.

Variable: Cytokines categories	Surgical intervention timing group		*p*- value
	Early group(≤48 h)	Delayed group (>48 h)	
Pro-inflammatory cytokines: Mean (SD)			
IL-1b			
PreOp	0.42 (0.73)	1.29 (4.97)	0.4003
PostOp	0.27 (0.18)	0.40 (0.36)	0.1165
Pre/postop difference	0.15 (0.70)	0.89 (4.97)	0.4746
IL-6			
PreOp	18.8 (32.6)	13.2 (24.7)	0.5097
PostOp	10.8 (23.9)	14.8 (28.8)	0.6050
Pre/postop difference	8.04 (38.4)	−1.62 (33.3)	0.3633
IL-8			
PreOp	24.2 (52.9)	15.2 (14.4)	0.4351
PostOp	43.0 (89.5)	85.7 (170.0)	0.2844
Pre/postop difference	−18.8 (80.9)	−70.5 (173.4)	0.1943
TNF-α			
PreOp	22.8 (16.9)	23.8 (15.6)	0.8304
PostOp	**19.0 (9.31)**	**25.3 (10.3)**	**0.0327**
Pre/postop difference	3.8 (15.7)	−1.49 (15.8)	0.2555
Anti-inflammatory cytokines: Mean (SD)			
IL-4			
PreOp	0.75 (1.05)	0.79 (0.99)	0.8796
PostOp	0.59 (0.45)	0.99 (0.91)	0.0519
Pre/postop difference	0.16 (1.20)	−0.20 (1.33)	0.3235
IL-10			
PreOp	6.75 (25.3)	3.70 (5.47)	0.5740
PostOp	2.11 (2.38)	3.40 (3.29)	0.1305
Pre/postop difference	4.65 (25.1)	0.31 (6.79)	0.4278
IL-13			
PreOp	0.22 (0.01)	0.26 (0.13)	0.1442
PostOp	0.24 (0.09)	0.24 (0.03)	0.7146
Pre/postop difference	−0.02 (0.09)	0.03 (0.13)	0.1684

Bold values: *p*-value < 0.05.

**Table 3:** Distribution of peri-operative mean serum levels of inflammatory cytokines according to post-resuscitation admission GCS and post-traumatic seizures (immediate and early PTS).

Variable	Post resuscitation -admission GCS			Immediate and early Post-traumatic seizures		
	9–13	14–15	*p*- value	No	Yes	*p*- value
Pro-inflammatory cytokines: Mean (SD)						
IL-1b						
PreOp	0.22 (0.06)	1.07 (4.05)	0.4745	0.37 (0.60)	2.24 (6.88)	0.1119
PostOp	0.23 (0.12)	0.37 (0.32)	0.1519	**0.29 (0.19)**	**0.48 (0.45)**	**0.0406**
Pre/postop difference	0.02 (0.15)	0.70 (4.05)	0.5484	0.09 (0.59)	1.76 (6.91)	0.1546
IL-6						
PreOp	5.90 (5.9)	19.6 (32.6)	0.1603	18.4 (31.6)	9.48 (18.2)	0.3634
PostOp	7.06 (4.8)	14.7 (30.2)	0.3874	10.7 (19.9)	18.9 (40.1)	0.3557
Pre/postop difference	−1.11 (7.3)	4.82 (41.5)	0.6270	7.67 (36.4)	−9.41 (32.9)	0.1577
IL-8						
PreOp	6.53 (4.56)	24.4 (44.3)	0.1726	22.3 (44.5)	12.7 (12.2)	0.4662
PostOp	13.6 (22.6)	81.2 (152.9)	0.1368	58.7 (105.4)	79.0 (204.7)	0.6597
Pre/postop difference	−7.04 (23.2)	−56.8 (154.9)	0.2773	−36.4 (102.7)	−66.3 (208.4)	0.5158
TNF-α						
PreOp	17.9 (14.3)	25.1 (16.5)	0.1878	21.4 (15.7)	28.9 (16.6)	0.1655
PostOp	**16.7 (11.6)**	**23.9 (9.15)**	**0.0313**	21.5 (9.36)	23.9 (12.7)	0.4950
Pre/postop difference	1.33 (12.4)	1.18 (17.0)	0.9777	−0.10 (14.0)	5.05 (20.3)	0.3354
Anti-inflammatory cytokines: Mean (SD)						
IL-4						
PreOp	0.40 (0.29)	0.90 (1.12)	0.1344	0.81 (1.22)	0.71 (0.44)	0.7487
PostOp	**0.34 (0.27)**	**0.94 (0.78)**	**0.0118**	**0.62 (0.49)**	**1.08 (0.98)**	**0.0362**
Pre/postop difference	0.06 (0.29)	−0.04 (1.46)	0.8161	0.19 (1.37)	−0.37 (0.99)	0.1455
IL-10						
PreOp	1.60 (1.34)	6.52 (21.2)	0.4284	6.32 (22.7)	3.40 (3.54)	0.6056
PostOp	**1.25 (0.72)**	**3.25 (3.20)**	**0.0379**	2.68 (3.17)	2.85 (2.46)	0.8448
Pre/postop difference	0.35 (0.92)	3.27 (21.5)	0.6429	3.64 (23.1)	0.55 (3.92)	0.5877
IL-13						
PreOp	0.22 (0.01)	0.25 (0.10)	0.3855	0.22 (0.01)	0.28 (0.14)	0.0510
PostOp	0.22 (0.004)	0.24 (0.08)	0.3569	0.24 (0.08)	0.23 (0.03)	0.6502
Pre/postop difference	0.001 (0.002)	0.01 (0.13)	0.8922	−0.02 (0.08)	0.04 (0.15)	0.0689

Bold values: *p*-value < 0.05.

**Table 4:** Distribution of peri-operative mean serum levels of inflammatory cytokines according to the clinical type of depressed skull fractures and intra-operative finding of a dural tear.

Variable	Type of depressed skull fractures			Intraoperative finding of a dura tear		
	Simple	Compound	*p*- value	No	Yes	*p*- value
Pro-inflammatory cytokines: Mean (SD)						
IL-1b						
PreOp	0.46 (0.69)	1.34 (5.21)	0.3963	0.44 (0.66)	1.45 (5.48)	0.3357
PostOp	0.38 (0.29)	0.28 (0.27)	0.2486	0.37 (0.29)	0.29 (0.28)	0.3430
Pre/postop difference	0.08 (0.72)	1.06 (5.18)	0.3445	0.07 (0.70)	1.17 (5.45)	0.2967
IL-6						
PreOp	17.2 (30.4)	14.7 (27.5)	0.7671	16.4 (29.4)	15.6 (28.8)	0.9280
PostOp	14.4 (33.9)	10.8 (12.0)	0.6449	14.7 (32.7)	9.99 (12.2)	0.5547
Pre/postop difference	2.84 (43.0)	3.90 (25.8)	0.9213	1.74 (41.6)	5.63 (26.5)	0.7200
IL-8						
PreOp	25.5 (50.5)	12.8 (14.5)	0.2688	25.7 (48.9)	11.2 (12.7)	0.2117
PostOp	57.1 (93.9)	72.4 (175.9)	0.7033	69.3 (114.9)	55.9 (163.9)	0.7424
Pre/postop difference	−31.5 (88.4)	−59.6 (178.8)	0.4853	−43.6 (113.2)	−44.8 (166.2)	0.9777
TNF-α						
PreOp	25.8 (16.3)	20.3 (15.7)	0.2480	24.6 (16.3)	21.5 (16.1)	0.5202
PostOp	24.2 (10.8)	19.5 (8.97)	0.1129	24.3 (10.5)	18.8 (9.14)	0.0685
Pre/postop difference	1.55 (15.5)	0.80 (16.5)	0.8748	0.24 (15.7)	2.65 (16.2)	0.6142
Anti-inflammatory cytokines: Mean (SD)						
IL-4						
PreOp	0.82 (1.02)	0.71 (1.01)	0.7228	0.79 (0.99)	0.75 (1.06)	0.8862
PostOp	0.90 (0.85)	0.65 (0.54)	0.2457	0.93 (0.83)	0.58 (0.52)	0.1090
Pre/postop difference	−0.08 (1.36)	0.06 (1.16)	0.6997	−0.14 (1.33)	0.17 (1.17)	0.4213
IL-10						
PreOp	7.58 (24.5)	2.40 (3.12)	0.3407	7.12 (23.6)	2.53 (3.26)	0.4052
PostOp	2.74 (3.08)	2.74 (2.75)	0.9965	2.92 (3.04)	2.47 (2.76)	0.6109
Pre/postop difference	4.84 (24.6)	−0.34 (3.96)	0.3459	4.20 (23.8)	0.05 (3.96)	0.4572
IL-13						
PreOp	0.24 (0.09)	0.25 (0.10)	0.8863	0.24 (0.08)	0.25 (0.10)	0.8146
PostOp	0.25 (0.09)	0.22 (0.01)	0.1969	0.25 (0.09)	0.22 (0.01)	0.2195
Pre/postop difference	0.01 (0.13)	−0.02 (0.09)	0.3770	−0.01 (0.12)	0.02 (0.10)	0.3577

**Table 5:** Distribution of peri-operative mean serum levels of inflammatory cytokines according to the neuro-radiological findings of increased ICP on brain CT (midline shift and status of basal cisterns).

Variable	Midline shift on CT Scan				Status of the basal cisterns		
	<5 mm	≥5 mm	*p*- value	Absent	Open	compressed	*p*- value
Pro-inflammatory cytokines: Mean (SD)							
IL-1b							
PreOp	0.90 (3.66)	0.29 (0.12)	0.7432	0.20 (0.08)	0.47 (0.75)	1.49 (5.47)	0.5868
PostOp	0.33 (0.29)	0.35 (0.31)	0.9053	0.41 (0.48)	0.31 (0.28)	0.34 (0.23)	0.7521
Pre/postop difference	0.57 (3.65)	−0.06 (0.19)	0.7352	−0.21 (0.47)	0.16 (0.77)	1.15 (5.45)	0.5810
IL-6							
PreOp	17.3 (29.9)	3.56 (4.74)	0.3691	6.78 (8.66)	14.4 (28.7)	21.0 (32.9)	0.5450
PostOp	13.1 (27.4)	8.99 (5.17)	0.7663	29.4 (52.3)	7.26 (5.46)	13.9 (28.9)	0.1833
Pre/postop difference	4.13 (37.5)	−5.43 (3.99)	0.6164	−22.6 (44.5)	7.14 (29.4)	7.07 (38.6)	0.1683
IL-8							
PreOp	20.9 (40.6)	8.97 (5.13)	0.5652	10.1 (8.27)	27.8 (55.5)	13.7 (10.5)	0.4213
PostOp	66.4 (140.9)	36.7 (39.4)	0.6792	10.5 (9.43)	84.14 (172.7)	57.4 (102.2)	0.4893
Pre/postop difference	−45.6 (141.3)	−27.8 (38.6)	0.8044	−0.43 (7.79)	−56.3 (175.8)	−43.7 (100.1)	0.6789
TNF-α							
PreOp	23.1 (16.6)	25.6 (10.6)	0.7678	24.5 (3.90)	25.3 (18.1)	20.6 (16.2)	0.6388
PostOp	21.3 (10.2)	30.7 (7.10)	0.0800	24.4 (14.9)	23.4 (9.0)	19.8 (10.1)	0.4627
Pre/postop difference	1.80 (16.2)	−5.03 (8.74)	0.4147	0.11 (17.6)	1.93 (15.7)	0.73 (16.2)	0.9570
Anti-inflammatory cytokines: Mean (SD)							
IL-4							
PreOp	0.79 (1.05)	0.53 (0.37)	0.6208	0.51 (0.35)	0.86 (1.12)	0.76 (1.05)	0.7641
PostOp	0.77 (0.75)	0.97 (0.56)	0.6087	0.98 (1.67)	0.77 (0.55)	0.74 (0.48)	0.7893
Pre/postop difference	0.02 (1.31)	−0.44 (0.30)	0.4897	−0.47 (1.48)	0.08 (1.29)	0.01 (1.20)	0.6450
IL-10							
PreOp	5.36 (19.1)	4.13 (5.40)	0.8989	0.89 (0.57)	2.01 (2.13)	10.4 (28.4)	0.2887
PostOp	2.57 (2.45)	4.52 (6.40)	0.2028	2.06 (1.95)	2.87 (2.79)	2.81 (3.36)	0.8311
Pre/postop difference	2.79 (19.2)	−0.40 (9.01)	0.7457	−1.16 (1.47)	−0.86 (2.85)	7.60 (28.6)	0.3079
IL-13							
PreOp	0.25 (0.23)	0.24 (0.09)	0.6663	0.23 (0.01)	0.24 (0.09)	0.25 (0.10)	0.8571
PostOp	0.24 (0.07)	0.23 (0.01)	0.6694	0.23 (0.01)	0.24 (0.03)	0.25 (0.10)	0.7236
Pre/postop difference	0.01 (0.12)	0 (0)	0.9316	0 (0)	0.01 (0.10)	0.001 (0.14)	0.9634

### 3.4 Changes of pre-and postoperative serum levels of inflammatory cytokines in regards to surgical timing of TBI patients

**Figure 3:**
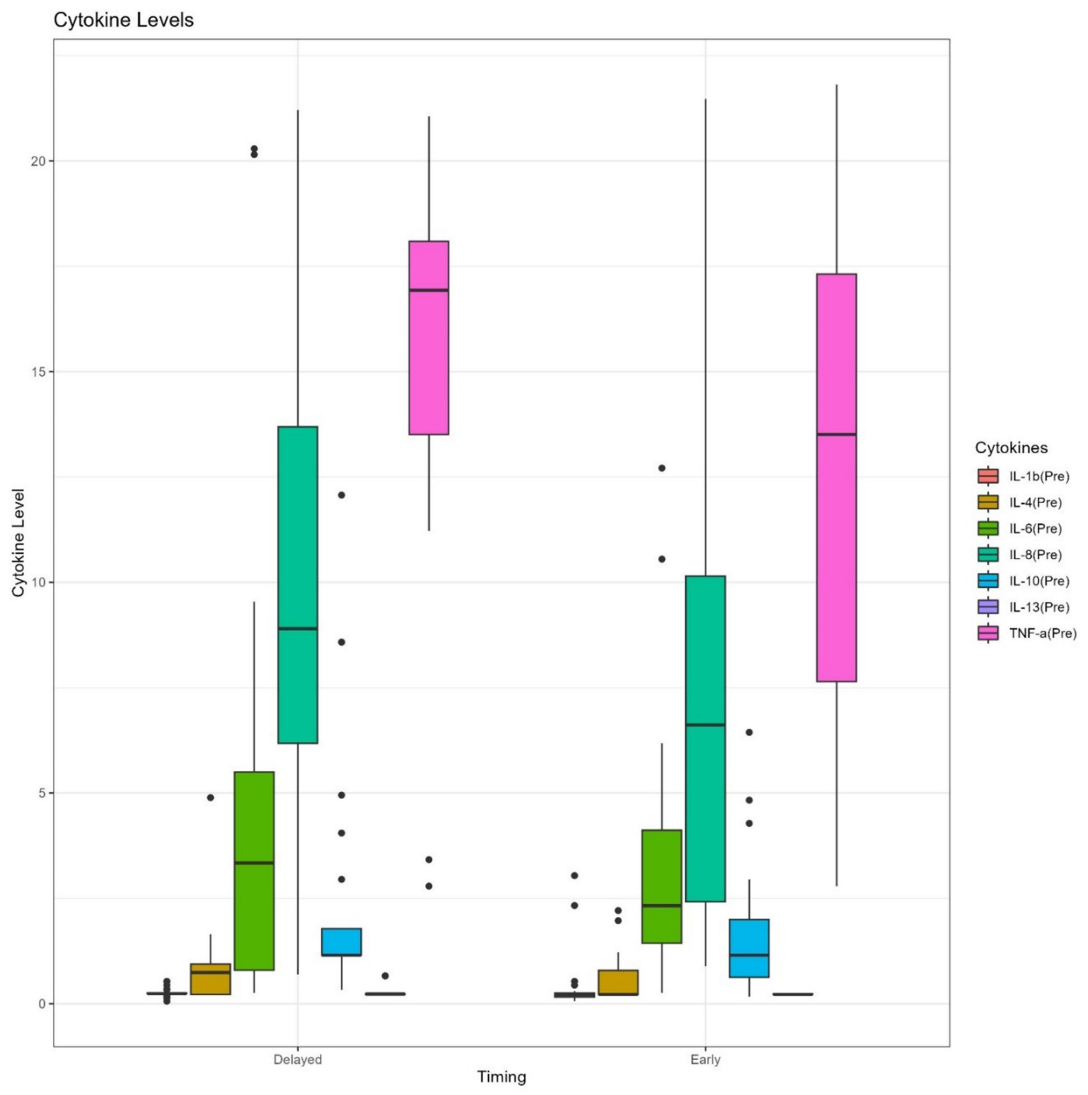
Box plots of pre-operative serum levels of pro- and anti-inflammatory cytokines between early and delayed surgical intervention groups.

**Figure 4:**
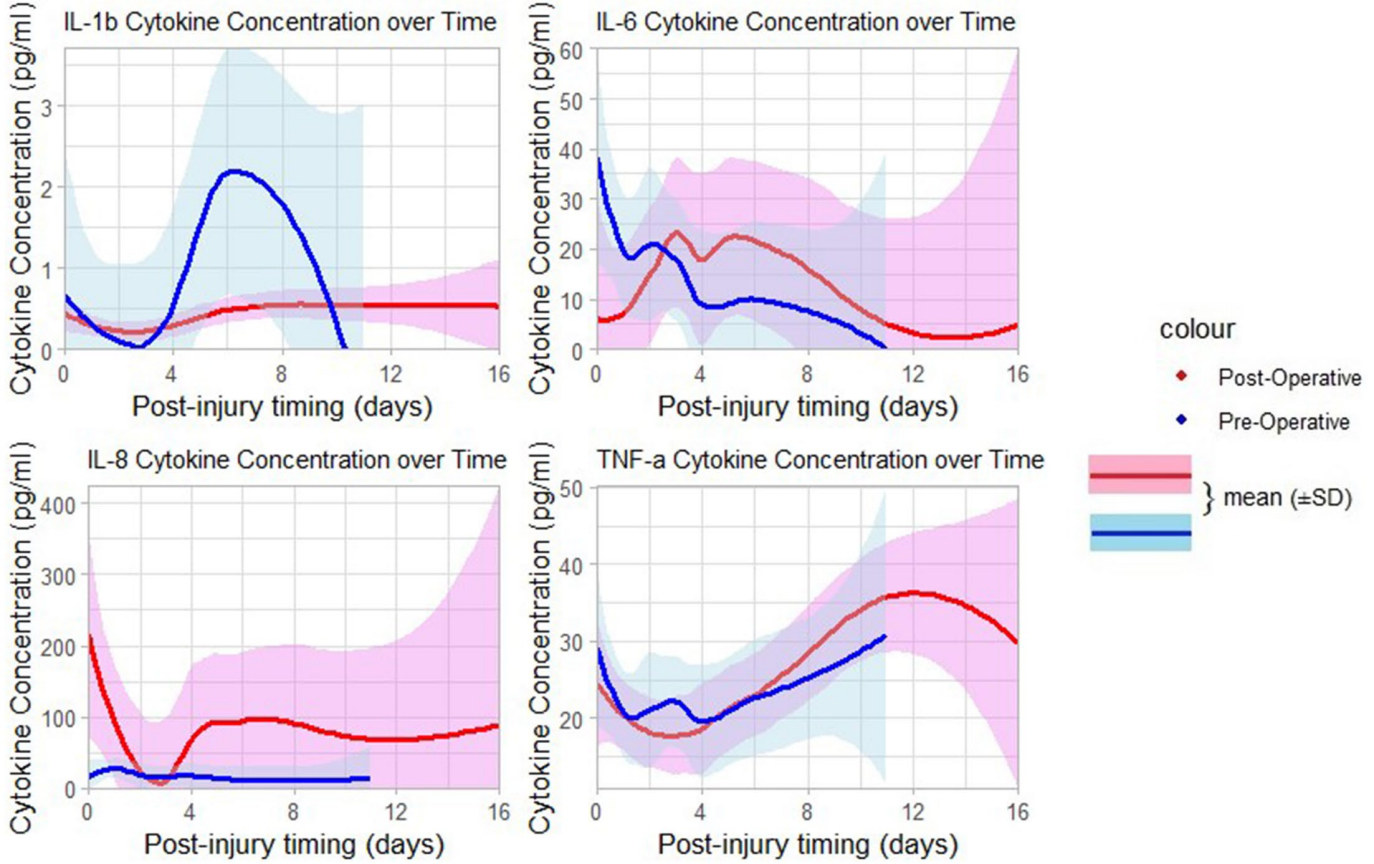
Curves of pre-and postoperative serum levels of pro-inflammatory cytokines over time post-injury.

**Figure 5:**
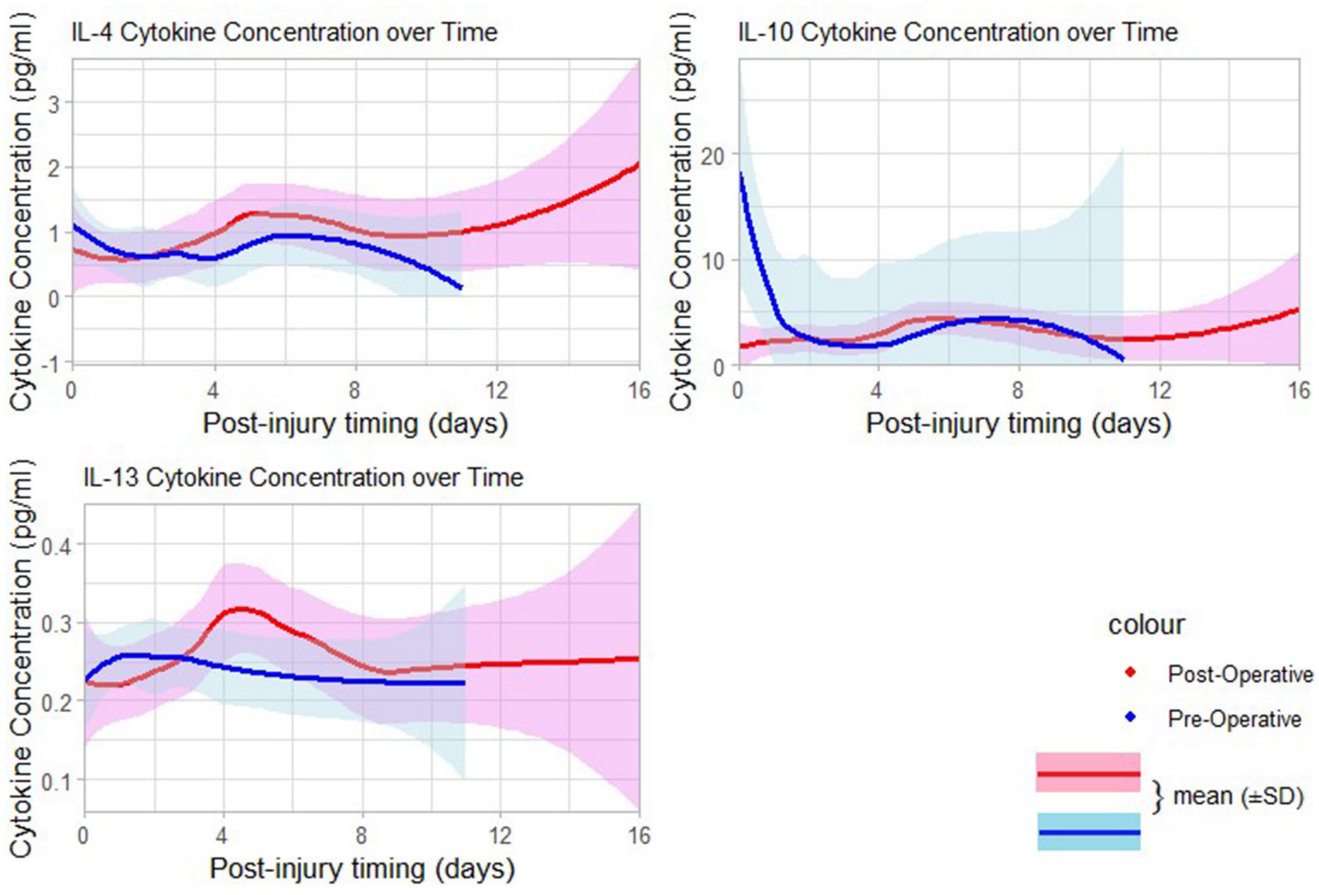
Curves of pre-and postoperative serum levels of anti-inflammatory cytokines time days post-injury.

The levels of TNF-*α* were detectable in the post-injury period, and significantly higher after surgery of the TBI patients who had delayed surgical treatment compared to those who had early surgery (*p* = 0.0327). The two groups of operative timing (early versus delayed) had relatively higher detectable serum concentrations of other types of inflammatory cytokines (IL-6, IL-8, and IL-10) but with no significant differences in changes between the two groups (Table 2). Patients who had delayed surgery (>48 h) had a higher mean of post-operative serum levels of the pro-inflammatory cytokines IL-6, and TNF-*α* in comparison to their pre-operative serum levels as shown in Figures 3–5, and Supplementary material S1.

### 3.5 Changes of pre-and postoperative serum levels of inflammatory cytokines in regards to the neurological presentations and findings

Regarding the post-resuscitation GCS, the serum levels of the pro-inflammatory TNF-α were significantly elevated postoperatively among TBI patients with a post-resuscitation admission GCS of 14–15 compared those with GCS of 9–13 (*p* = 0.0313); the serum levels of the anti-inflammatory cytokines IL-4 and IL-10 were also elevated considerably postoperatively among TBI patients with post-resuscitation admission GCS of 14–15 compared those with GCS of 9–13 (*p* = 0.0118 and *p* = 0.0379 respectively). No statistically significant differences were noted for other inflammatory cytokines (Table 3). The levels of the pro-inflammatory cytokine IL-1b and the anti-inflammatory cytokine IL-4 were significantly elevated postoperatively among TBI patients who presented with immediate and early PTS than those who did not present with PTS (*p* = 0.0382 and *p* = 0.0362 respectively); no statistically significant differences were noted for other inflammatory cytokines (Table 3). The serum levels of pre-and postoperative pro- and anti-inflammatory cytokines (IL-1b, IL-6, TNF-a, and IL 10) were higher among TBI patients with DSF regardless of the clinical types (simple or compound), and the presence or not of an intraoperative finding of dural tear but without a significant statistical difference between the groups (Table 4).

### 3.6 Changes of pre-and postoperative serum levels of inflammatory cytokines in regards to the neuro-radiological findings (features of increased ICP on brain CT)

There were no statistically significant differences noted for the pre-and postoperative serum inflammatory cytokines (Table 5). However, patients with MLS <5 mm had relatively higher serum levels of IL-6 in comparison to those with MLS ≥ 5 mm but without statistically significant differences.

### 3.7 Changes of pre-and postoperative serum levels of inflammatory cytokines in regards to patients’ outcomes

Changes between pre- and post-operative serum concentration of the anti-inflammatory IL-10 was found statistically significantly higher among patients who developed SSI in comparison to those who did not develop [11.56 (35.5) pg./mL versus −0.58 (4.02) pg./mL, *p* = (0.0489)] (Table 6). No significant changes in serum levels of inflammatory cytokines were noted neither for hospital outcomes 3 months after surgery nor overall eGOS after 6 months.

**Table 6:** Distribution of peri-operative mean serum levels of inflammatory cytokines according to patients’ management outcomes (surgical site infection and hospital outcomes).

Variable	Surgical site infection				Hospital outcomes after surgery		
	No	Yes	*p*- value	Improved	Prolonged stay/Re-operation	Died	*p*- value
Pro-inflammatory cytokines: Mean (SD)							
IL-1b							
PreOp	1.01 (3.94)	0.27 (0.09)	0.5594	1.01 (3.94)	0.27 (0.10)	0.24 (0)	0.8449
PostOp	0.35 (0.31)	0.27 (0.16)	0.4243	0.35 (0.31)	0.27 (0.17)	0.24 (0)	0.064
Pre/postop difference	0.65 (3.94)	0.003 (0.17)	0.6033	−0.65 (3.94)	0.003 (0.20)	0 (0)	0.8750
IL-6							
PreOp	16.5 (28.2)	14.5 (32.7)	0.8430	16.5 (28.2)	16.0 (34.3)	0.79 (0)	0.8700
PostOp	14.5 (29.4)	6.56 (4.22)	0.4047	14.5 (29.4)	6.46 (4.47)	7.49 (0)	0.7090
Pre/postop difference	2.07 (37.0)	7.90 (33.1)	0.6542	2.07 (37.0)	9.52 (34.7)	−6.70 (0)	0.8292
IL-8							
PreOp	22.6 (43.5)	9.79 (5.85)	0.3635	22.6 (43.5)	9.77 (6.20)	10.0 (0)	0.6649
PostOp	73.1 (150.4)	29.8 (36.8)	0.3743	73.1 (150.4)	32.0 (38.2)	9.51 (0)	0.6688
Pre/postop difference	−50.6 (151.5)	−20.0 (33.7)	0.5323	−50.6 (151.5)	−22.3 (34.9)	0.49 (0)	0.8146
TNF-α							
PreOp	23.3 (16.9)	23.2 (13.6)	0.9830	23.3 (16.9)	23.1 (14.4)	24.0 (0)	0.9986
PostOp	22.3 (10.1)	21.4 (11.3)	0.8034	22.3 (10.1)	22.3 (11.7)	13.3 (0)	0.6962
Pre/postop difference	−1.05 (17.2)	−1.84 (9.65)	0.8891	−1.05 (17.2)	0.87 (9.70)	10.6 (0)	0.8394
Anti-inflammatory cytokines: Mean (SD)							
IL-4							
PreOp	0.88 (1.13)	0.45 (0.30)	0.2039	0.86 (1.11)	0.45 (0.35)	0.53 (0)	0.5459
PostOp	0.83 (0.78)	0.66 (0.59)	0.4840	0.82 (0.76)	0.67 (0.66)	0.53 (0)	0.8055
Pre/postop difference	0.05 (1.44)	−0.21 (0.46)	0.5496	0.03 (1.41)	−0.22 (0.51)	0 (0)	0.8703
IL-10							
PreOp	2.32 (2.76)	13.8 (35.7)	0.0599	5.61 (20.4)	4.27 (7.70)	1.15 (0)	0.9576
PostOp	2.90 (3.22)	2.27 (1.69)	0.5200	2.84 (3.16)	2.30 (1.86)	2.95 (0)	0.8832
Pre/postop difference	**−0.58 (4.02)**	**11.56 (35.5)**	**0.0489**	2.77 (20.6)	1.97 (8.08)	1.80 (0)	0.9674
IL-13							
PreOp	0.25 (0.10)	0.23 (0.01)	0.4063	0.25 (0.10)	0.22 (0.01)	0.23 (0)	0.7405
PostOp	0.24 (0.08)	0.23 (0.03)	0.7792	0.24 (0.07)	0.24 (0.04)	0.23 (0)	0.9843
Pre/postop difference	0.01 (0.13)	−0.01 (0.03)	0.6253	−0.01 (0.13)	0.01 (0.04)	0 (0)	0.8685

Bold values: *p*-value < 0.05.

### 3.8 Changes of pre-and postoperative serum levels of inflammatory cytokines in relation to the post-traumatic seizure outcomes events

Regarding the late PTS up to 6 months, there were no statistically significant differences noted for the pre-and postoperative serum inflammatory cytokines (Table 7). However, when considering a cumulating incidence of all seizure events in a patient (immediate, early, and late PTS up to 6 months), we found that the changes in pre-and postoperative serum concentration of the pro-inflammatory IL-1b and the anti-inflammatory IL-4 were statically significantly different among those who experienced PTS in comparison to those who did not {respectively: 0.27 (0.17) pg./mL versus 0.45 (0.40) pg./mL *p* = 0.0382; and 0.62 (0.49) pg./mL versus 1.08(0.98) pg./mL, *p* = 0.0362} (Table 7).

**Table 7:** Distribution of peri-operative mean serum levels of inflammatory cytokines according to post-traumatic seizure outcomes events (late PTS up to 6 months and cumulative PTS events).

Variable	Late Post-traumatic seizures (up to 6 months)			Cumulative PTS events (immediate, early, or late)		
	No	Yes	*p*- value	No	Yes	*p*- value
Pro-inflammatory cytokines; Mean (SD)						
IL-1b						
PreOp	0.90 (3.66)	0.29 (0.12)	0.7432	1.18 (4.37)	0.26 (0.10)	0.3933
PostOp	0.33 (0.29)	0.35 (0.31)	0.9053	**0.27 (0.17)**	**0.45 (0.40)**	**0.0382**
Pre/postop difference	0.57 (3.65)	−0.06 (0.19)	0.7352	0.91 (4.34)	−0.19 (0.39)	0.3059
IL-6						
PreOp	17.3 (29.9)	3.56 (4.74)	0.3691	17.1 (29.8)	14.3 (27.8)	0.7526
PostOp	13.1 (27.4)	8.99 (5.17)	0.7663	13.0 (23.0)	12.4 (31.9)	0.9372
Pre/postop difference	4.13 (37.5)	−5.43 (3.99)	0.6164	4.10 (34.1)	1.93 (40.01)	0.8452
IL-8						
PreOp	20.9 (40.6)	8.97 (5.13)	0.5652	20.9 (47.9)	17.9 (13.7)	0.8052
PostOp	66.4 (140.9)	36.7 (39.4)	0.6792	55.5 (106.9)	78.8 (177.6)	0.5766
Pre/postop difference	−45.6 (141.3)	−27.8 (38.6)	0.8044	−34.6 (102.5)	−60.8 (−154.6)	0.5294
TNF-α						
PreOp	23.1 (16.6)	25.6 (10.6)	0.7678	22.9 (19.2)	23.9 (8.61)	0.8617
PostOp	21.3 (10.2)	30.7 (7.10)	0.0800	21.7 (9.13)	22.8 (12.2)	0.7298
Pre/postop difference	1.80 (16.2)	−5.03 (8.74)	0.4147	1.29 (17.3)	1.07 (13.1)	0.9635
Anti-inflammatory cytokines; Mean (SD)						
IL-4						
PreOp	0.79 (1.05)	0.53 (0.37)	0.6208	0.81 (1.22)	0.71 (0.44)	0.7487
PostOp	0.77 (0.75)	0.97 (0.56)	0.6087	**0.62 (0.49)**	**1.08 (0.98)**	**0.0362**
Pre/postop difference	0.02 (1.31)	−0.44 (0.30)	0.4897	0.19 (1.37)	−0.37 (0.99)	0.1455
IL-10						
PreOp	5.36 (19.1)	4.13 (5.40)	0.8989	6.32 (22.7)	3.40 (3.54)	0.6056
PostOp	2.57 (2.45)	4.52 (6.40)	0.2028	2.68 (3.17)	2.85 (2.46)	0.8448
Pre/postop difference	2.79 (19.2)	−0.40 (9.01)	0.7457	3.64 (23.1)	0.55 (3.92)	0.5877
IL-13						
PreOp	0.25 (0.23)	0.24 (0.09)	0.6663	0.22 (0.01)	0.28 (0.14)	0.0510
PostOp	0.24 (0.07)	0.23 (0.01)	0.6694	0.24 (0.08)	0.23 (0.03)	0.6502
Pre/postop difference	0.01 (0.12)	0 (0)	0.9316	−0.02 (0.08)	0.04 (0.15)	0.0689

Bold values: *p*-value < 0.05.

### 3.9 Relationship between the duration of general anesthesia and duration of surgery on the post-operative serum levels of pro- and anti-inflammatory cytokines

On the visualization of the curves of post-operative concentration of pro- and anti-inflammatory cytokines and the duration of the general anesthesia and the duration of surgery, there was a general trend in the increase of serum concentration of those cytokines in response with the prolongation of the duration of general anesthesia and surgery among patients (Supplementary materials S2, S3).

### 3.10 Factors associated with the changes of serum levels of inflammatory cytokines in the peri-operative period

Multivariate analysis was performed to evaluate associations between the changes of pre-and postoperative serum concentration of four contributing inflammatory cytokines (2 pro-inflammatory cytokines, IL-6, TNF-*α*, and two anti-inflammatory cytokines, IL-4, IL-10) in regarding the different universal confounders, clinic-radiological variables, and outcomes variables of study participants operated with DSF. The average change in the serum concentration of the anti-inflammatory IL-4 between the pre-and postoperative samples decreased by 0.87 units among participants with pre-operative hemoglobin of 10–12 g/dL compared to those with hemoglobin concentration > 12 g/dL (adjusted Coefficients = aCoef, 0.87; 95%CI, 0.01: 1.74). There was no significant association in the average change of anti-inflammatory IL-4 cytokine for those with a hemoglobin concentration of <10 g/dL (aCoef, 0.07; 95%CI, 1.06: 1.20). (Table 8). The average change in the serum concentration of the pro-inflammatory TNF-*α* between the pre-and postoperative samples increased by 1.12 units among participants with a history of PTS (immediate, early, and late seizure up to 6 months) compared to those who never experienced PTS up to 6 months (aCoef, −1.12; 95%CI, −1.80: −0.45, *p* = 0.01). The average change in the serum concentration of the pro-inflammatory TNF-*α* between the pre-and postoperative samples decreased by 1.02 units among participants with TBI associated with long bone fracture compared to those without associated long bone fractures (aCoef, 1.02; 95%CI, 0.24: 1.81, *p* = 0.05). Patients who presented with a focal neurological deficit (hemiparesis, monoparesis, and cranial nerve palsy) have an increased average change from pre- to postoperative serum concentration of the pro-inflammatory TNF-α by −0.64 units, of the pro-inflammatory IL-6 by 1.75 units, and by the anti-inflammatory IL-4 by −1.04 units compared to those who did not present with a focal neurological deficit{TNF-α (aCoef, −0.64; 95%CI, −1.25: -0.04), IL-6, (aCoef, −1.75; 95%CI, −3.32: −0.18), and IL-4 (aCoef, −1.04, 95%CI, −2.00: −1.08), p = 0.05} (Table 8). The average change in the serum concentration of the anti-inflammatory IL-10 between the pre-and postoperative samples increased by 1.07 units among participants with DSF with underlying significant extra-axial hemorrhage compared to those with DSF without underlying significant extra-axial hemorrhage (aCoef, −1.07, 95%CI, −2.02: −0.10, *p* = 0.05).

**Table 8:** Association between independent variables and change between the pre/postop mean serum levels of inflammatory cytokines (TNF-α, IL-6, IL-4, and IL-10).

Variable	LogTNF-α	LogIL6	LogIL4	LogIL10
	aCoef (95%CI)	aCoef (95%CI)	aCoef (95%CI)	aCoef (95%CI)
Age (Years)	0.024 (−0.002: 0.05)	−0.001 (−0.07: 0.07)	−0.004 (−0.05: 0.04)	0.002 (−0.05: 0.05)
Sex of the patients				
Female	1	1	1	1
Male	0.65 (−0.68: 1.97)	−1.20 (−4.64: 2.24)	0.29 (−1.90: 2.47)	0.03 (−2.34: 2.39)
Surgical timing				
Early	1	1	1	1
Delayed	0.14 (−0.47: 0.74)	0.65 (−0.86: 2.16)	0.40 (−0.50: 1.29)	0.01 (−1.01: 1.03)
ASA classification				
Normal health	1			
Mild systemic disease	0.56 (−0.57: 1.68)			
Severe systemic disease	0.73 (−0.52: 1.99)			
Mechanism of injury				
Assault				
Passenger motor cycle RTC				
Others				
Surgical site infection				
No	1	1	1	1
Yes	−0.12 (−0.84: 0.58)	−0.02 (−1.55: 1.52)	0.42 (−0.53: 1.37)	0.28 (−0.81: 1.36)
Hemoglobin (g/dl)				
>12	1	1	**1**	1
10–12	−0.39 (−0.91: 0.11)	0.47 (−0.88: 1.81)	**0.87 (0.01: 1.74)** *	0.06 (−0.94: 1.06)
<10	0.19 (−0.63: 1.01)	−0.88 (−2.71: 0.96)	0.07 (−1.06: 1.20)	−1.13 (−2.42: 0.16)
Intraoperative dural tear				
No	1	1	1	
Yes	−0.50 (−1.64: 0.64)	−0.02 (−3.07: 3.02)	−1.69 (−3.59: 0.22)	
Early PTS				
No	1	1	1	1
Yes	−0.08 (−0.64: 0.47)	0.47 (−0.91: 1.85)	0.01 (−0.84: 0.85)	0.55 (−0.43: 1.52)
Cumulative PTS events				
No	**1**	1	1	1
Yes	−1.12 (−1.80: −0.45)**	−0.84 (−2.57: 0.90)	0.14 (−0.88: 1.16)	−0.56 (−1.76: 0.63)
Post-resuscitation admission GCS				
9–13	1	1	1	1
14–15	−0.14 (−0.86: 0.58)	−1.21 (−2.97: 0.55)	−0.57 (−1.69: 0.55)	0.67 (−0.43: 1.78)
Long bone fractures				
No	**1**			
Yes	1.02 (0.24: 1.81) *			
Neurological focal sign				
No deficit	**1**	**1**	**1**	
Deficit	−0.64 (−1.25: −0.04) *	**−1.75 (−3.32: −0.18)** *	−1.04 (−2.00: −1.08) *	
Location of DSF depressed skull				
Frontal				
Frontoparietal				
Parieto				
Temporal				
Presence of contusion				
No				
Yes				
Extra axial hemorrhage				
No				**1**
Yes				−1.07 (−2.02: −0.10) *
Basal cisterns in radiological finding				
Absent			1	
Open			−0.42 (−1.55: 0.71)	
Compressed			0.26 (−0.96: 1.49)	
Midline shift 5 mm				
No	1	1		
Yes	0.77 (−0.03: 1.57)	1.79 (−0.28: 3.86)		
Type of depressed skull				
Simple	1	1	1	1
Compound	0.39 (−0.80: 1.59)	0.96 (−2.03: 3.96)	1.35 (−0.59: 3.28)	1.03 (−1.16: 3.22)
Dural tear				
No	1	1	1	1
Yes	−0.50 (−1.64: 0.64)	−0.02 (−3.07: 3.02)	−1.69 (−3.59: 0.22)	−0.28 (−2.50: 1.93)

aCoef. = Adjusted Coefficients *p < 0.05 **p < 0.01 ***p < 0.001. Bold values: *p*-value < 0.05.

## 4 Discussion

This study set out to elucidate the relationship between the timing of surgery and changes in serum levels of perioperative inflammatory cytokines to optimize TBI management in LMIC. It has also explored the association between inflammatory cytokines and clinical-radiological parameters.

### 4.1 Demographics, clinical-radiological presentations, and management

The two groups of interest (surgical intervention ≤48 h versus >48 h) had similar demographics (age, sex, and other injury factors). Most of the participants were young adults with a median age of 25.5 years as reported in the literature (40, 41). Half of them were victims of assaults from criminal activities in the periphery of major cities of Uganda usually late at night or before sunrise. This phenomenon is known as boda-boda (motorcycle) thieves’ assault, in which motorcycle taxi men, passengers (or customers) are hit on the convexity of the head with a metallic bar or hammer by unknown thieves before being robbed, and left unattended in the street. This results in a localized surface of DSF with potential underlying intracranial damage. Several studies done in LMIC on DSF reported assault as the principal mechanism (42). Most of them have a higher post-resuscitation GCS and are relatively stable. This could also justify the delay in neuro-imaging assessment and referral to the neurosurgical centers since in Uganda, unconscious patients tend to be referred in priority for urgent brain CT scans compared to those who are relatively awake. Perioperatively, participants were hemodynamically stable with a mean pre-operative hemoglobin rate of 12.2 (±2.3) g/dL, ASA classification <3 in 84.2%, and more than 50% of them had no features of brain edema on the initial CT scan. They had standardized acute trauma care and resuscitation in the perioperative management; they received prophylaxis antibiotics, anti-epileptic treatment, prophylactic intraoperative antibiotics as recommended for infection prevention (43), and other treatments. We did not include these parameters in our analysis and we bear in mind that they could also contribute slightly to the change of inflammatory response in any direction (reducing or increasing), but probably in an equal manner.

### 4.2 Serum inflammatory cytokines levels, the timing of surgery of TBI, and factors associated

The association between the pre-and post-operative changes of pro- and anti-inflammatory cytokines based on the different surgical timing groups is also a novel aspect of these findings. Previous studies done on TBI demonstrated trending changes in both cerebrospinal fluid (CSF) and serum cytokines as a result of brain insult in small animals and observational human studies (44–47). In our study, the serum levels of major inflammatory cytokines (IL-6, TNF-*α*) following surgery done within 48 h show a downward trend when compared to the surgery done after 48 h; this supports the hypothesis that surgical procedure and anesthetic agents may contribute to the reduction of the amplitude of the response of the systemic inflammation in TBI patients, thus, contribute to the reduction of neuro-inflammatory processes, as previously observed by in one observational study (48). In visualization of the curves of mean serum levels of inflammatory cytokines following days of injury (Figures 4, 5), the initially produced pro-inflammatory cytokines tend to decrease in amplitude on the second day of injury before rising again; this trend is seen among patients operated in beyond 48 h. This supports the findings of the study done by Frugier et al. about the *in-situ* detection of inflammatory cytokines in post-mortem human brain between 40 and 129 h after TBI where mRNA expression of pro-inflammatory (IL-1b, IL-6, IL-8, and TNF-α) was significantly increased in delayed hours (37).

Regarding IL-6, similar findings were reported in a study done by Osuka et al. in Japan on the change of the serum concentrations of inflammatory cytokines following non-TBI neurosurgical procedures among 70 patients; the serum levels of IL-6 were elevated following surgery, with a peak in the postoperative day one, and gradually decreasing after; they hypothesized that IL-6 is an early marker of tissue damage of the postoperative stress (49). Similar findings of raised IL-6 postoperatively were reported in a study done on 31 blunt trauma orthopedics procedures there was an increase of serum IL-6 within 24 h of surgery, and a decrease gradually by 72 h (50). Another study analyzing *in-situ* detection of intracerebral pro-and anti-inflammatory cytokines’ expression from biopsies of contused brain tissue postoperatively was done among 12 TBI patients between three to 5 days following the trauma (38); they found that in surgery done in 24 h after trauma, there was a strong expression of both the pro- (IL-1β, IL-6, IFN-*γ*) and anti-inflammatory cytokines (IL-4), and surgery done by three to 5 days after trauma, the anti-inflammatory IL-4 had a significant lower expression.

In our study, the postoperative increase in serum levels of the anti-inflammatory IL-4 and IL-10 were also associated with a higher post-resuscitation admission GCS of 14–15; this is probably due to a neuroprotective role of anti-inflammatory in down-regulating the acute inflammatory response by suppressing the unnecessary action of pro-inflammatory cytokines and promoting the synthesis of neurotrophic factors and healing in patients with minor TBI and survivors (16, 51). In contrast, although we did not include patients with GCS <9, it was demonstrated that increased serum and CSF levels of the pro-inflammatory IL-8 and TNF-*α* have a relationship with an impending increased ICP and cerebral hypoperfusion in patients with low GCS (52). The change of serum levels of the pro-inflammatory IL-1b and the anti-inflammatory IL-4 were associated with the history of post-traumatic seizure at any time before or after surgery up to 6 months. The two cytokines may play antagonist roles, but we bear in mind that the clinical findings of seizure may have been unrevealed in many patients since all patients received systematic anti-seizure prophylaxis from admission up to a minimum of 2 weeks from surgery, and we did not perform for continuous electroencephalogram in the initial admission. Indeed, a study done on TNF-*α* in TBI showed a strong association of acutely elevated levels of TNF-α in PTS patients as compared to non-PTS patients (53). Looking at the brain CT scan findings that are frequently encountered in head injury, especially the ones indicating features of increased ICP, patients with MLS <5 had relatively higher serum levels of IL-6 in comparison to those with MLS ≥ 5 but without significant differences. About 84% of participants had underlying contusions on brain CT scans. This can justify the quasi-detection of serum cytokines among our participants as evidence of neuronal damage postulated by Edwards KA et al. in their study on serum cytokines between mild TBI and non-TBI using both brain CT and MRI scan, where they found elevated serum levels of IL-6, TNF-*α*, and VEGF among mild TBI within 24 h of injury (36). We also found that SSI was associated with the changes in pre-and post-operative serum concentration of the anti-inflammatory IL-10, but for the other cytokines, we did not find a significant difference. However, without including the pre-operative serum levels, another study was done by Nannan Zhang et al. looked at the postoperative changes on day one, day three, and day five among 120 operated TBI patients within 3 days regardless of GCS, consisting of 60 cases who developed postoperative intracranial infections versus a control group of 60 who did not (39); they found that the severity of the postoperative intracranial infection was correlated with an increase of serum levels of an extended panel of inflammatory cytokines (IL-1β, IL-2, IL-6, IL-8, TNF-*α*, IFN-*γ*); they did not analyze the serum level of IL-10. Whereas, in our study, we included pre- and postoperative samples of operated mild–moderate TBI patients within and beyond 48 h. We believe that their methods (including only postoperative samples, patients’ selection before surgery, type of procedures, TBI severity classification, operational definition of SSI, etc.) might largely be different from ours as well as we cannot ascertain the matching of the two groups from the available published literature.

The dynamic of cytokines production remains the cornerstone of understanding the impact of the timing of TBI surgery on their outcomes, especially by the attempt to model clinical and biological parameters. The history of PTS was associated with a postoperative increase in TNF-*α* (*p* = 0.01), the hemoglobin of 10–12 with a postoperative decrease of IL-4 (*p* = 0.05), and the presence of focal neurological deficit was associated with a significant postoperative increased of TNF-α, IL-6, and IL-4 (p = 0.05). The presence of extra-axial hemorrhage was associated with a postoperative increase of IL-10 (p = 0.05). From our study, we can stipulate that the timing of the neurosurgical procedures among mild–moderate TBI associated with reduced hemoglobin concentration, focal neurological deficit, intra-cranial extra-axial hemorrhage, and the administration of general anesthetic agents have significant implications on the dynamics of the inflammatory cytokines productions, affecting the hemostatic physiology, the immune response, and the worsening the neuro-inflammation. Our findings demonstrated a relationship between the timing of surgery in TBI patients and the postoperative inflammatory response as seen by changes in serum concentration of pro- and anti-anti-inflammatory cytokines. Delayed surgical intervention after 48 h of injury was associated with increased levels of TNF-*α* and IL-6, suggesting an exacerbated inflammatory reaction. Clinical factors such as neurological deficits, post-traumatic seizures, reduced hemoglobin, and extra-axial hemorrhage further contribute to this response, potentially impacting patient outcomes. This highlights the importance of timely surgical intervention in managing TBI with a hypothesis that there is a potential need for biological monitoring of the inflammatory responses through cytokines serum levels in patients undergoing delayed surgery. In addition, emphasis on potential clinical implications on therapeutic pathways through regulations of the serum levels of pro- and anti-inflammatory cytokines. Our study can extrapolate the therapeutic idea of promoting IL-4 to down-regulate the neuro-inflammation induced by IL-6 and TNF-α in case of TBI surgery done beyond 48 h of injury and improving the pre-operative hemoglobin, and efficient seizure control.

Through this understanding of the patient’s clinical and biological parameters, we found that 48 h could be an acceptable window between injury and surgical intervention. Poor outcomes after surgical intervention beyond 48 h in TBI patients such as SSI, delayed PTS, and expansion of the extra-axial hemorrhage may be correlated with a significant change in serum levels of pro- and anti-inflammatory cytokines, and their possible imbalance to maintain hematologic and immunologic homeostasis. We hypothesized that this could be caused by a second pic of the neuro-inflammatory process from a possible ongoing worsening rise of ICP through brain edema.

### 4.3 Study limitation

The allocation of surgical timing might have been affected by the inherent decision of the neurosurgical team; in addition, the study was conducted just after the first wave of COVID-19 in Uganda, during which there was a delay in patients’ referral in term of mobility restriction, the readiness of the brain CT scan, and the readiness of operating theaters. We believe that our wide exclusion criteria might have counterbalanced these limitations. We had to rely on the estimated time that the patient (or his next-of-kin) last recalled being fully awake before the injury and reported the groups in brackets of days. Lastly, our study was done by measuring mainly the serum levels of inflammatory cytokines from at least two-time points during the perioperative period. Additional sampling time points and analysis of other inflammatory mediators (other cytokines, chemokines, and acute-phase proteins) could have provided a more comprehensive understanding of the inflammatory response following TBI surgery. We used the post-resuscitation admission GCS as the gold standard for TBI severity, yet GCS is dynamic. We also acknowledge the influence of individual patients’ inflammatory responses based on their previous unidentified exposure to inflammatory stimuli. This may affect the profile and extent of the serum cytokines. Our study was done in a single center with a relatively small sample size, which may limit the generalizability of the results.

## 5 Conclusion

The surgical intervention done beyond 48 h of injury for patients with mild–moderate TBI has an increased postoperative response of the serum levels of pro-inflammatory cytokines, and eventually prolongs the impact of neuro-inflammation postoperatively. Factors like neurological deficit, post-traumatic seizure, reduced hemoglobin rate, and extra-axial intracranial hemorrhage contribute significantly to this response. Future research with larger cohorts of TBI patients should focus on elucidating the impact of surgical timing on the change of targeted serum levels of inflammatory cytokines with greater precision and understanding the underlying mechanisms driving the observed differences and factors associated with mitigating their adverse effects of the surgical outcomes.

## Data Availability

The original contributions presented in the study are included in the article/Supplementary material, further inquiries can be directed to the corresponding author.
