# Supplementary figures and images for: Changes in serum concentration of perioperative inflammatory cytokines following the timing of surgery among mild–moderate traumatic brain injury patients and factors associated
Source: Front Neurol. 2024 Dec 18;15:1484742. doi: 10.3389/fneur.2024.1484742 (PMC11688224; doi:10.3389/fneur.2024.1484742)

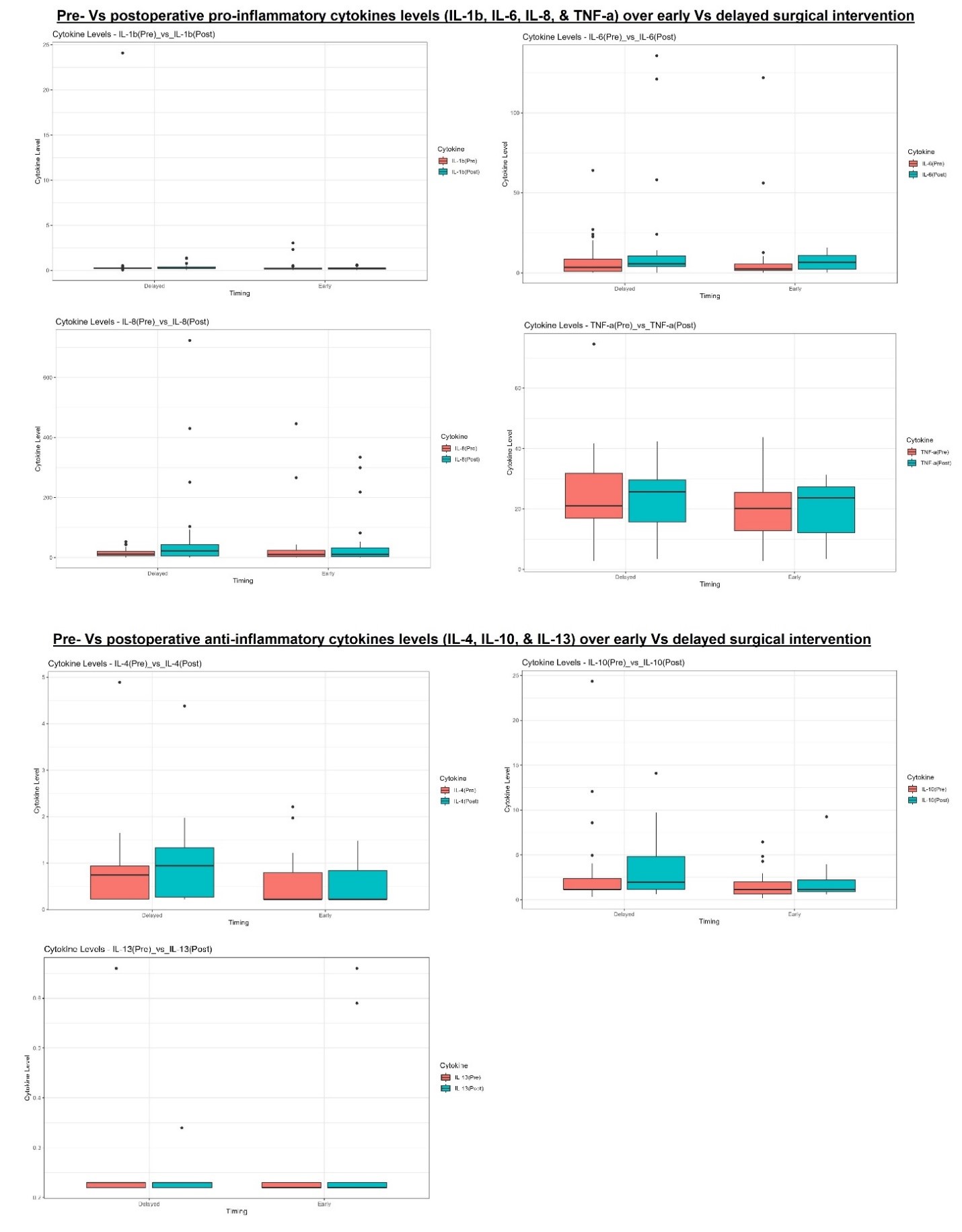

Supplement: Supplementary file 1 [file Image_1.JPEG]

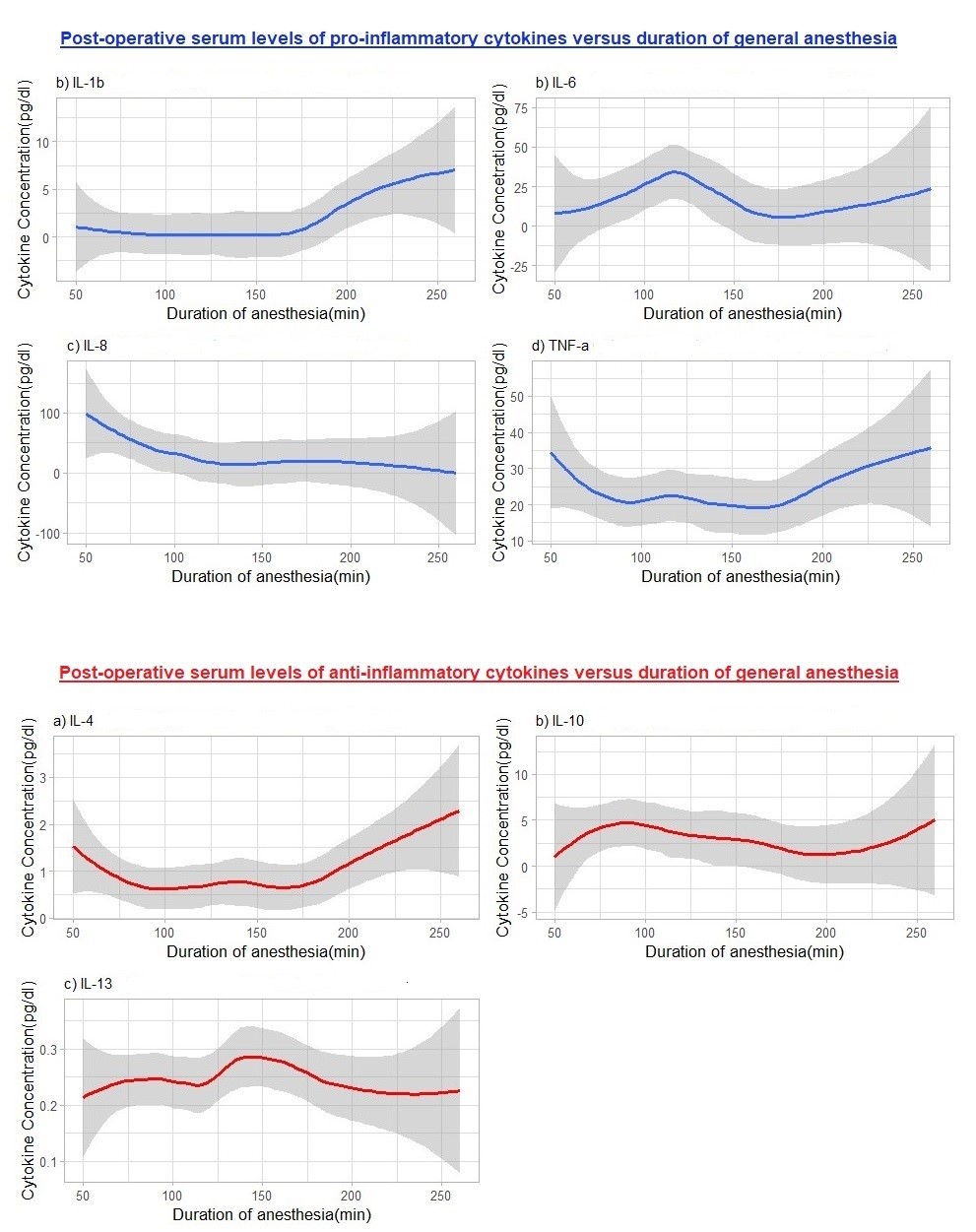

Supplement: Supplementary file 2 [file Image_2.JPEG]

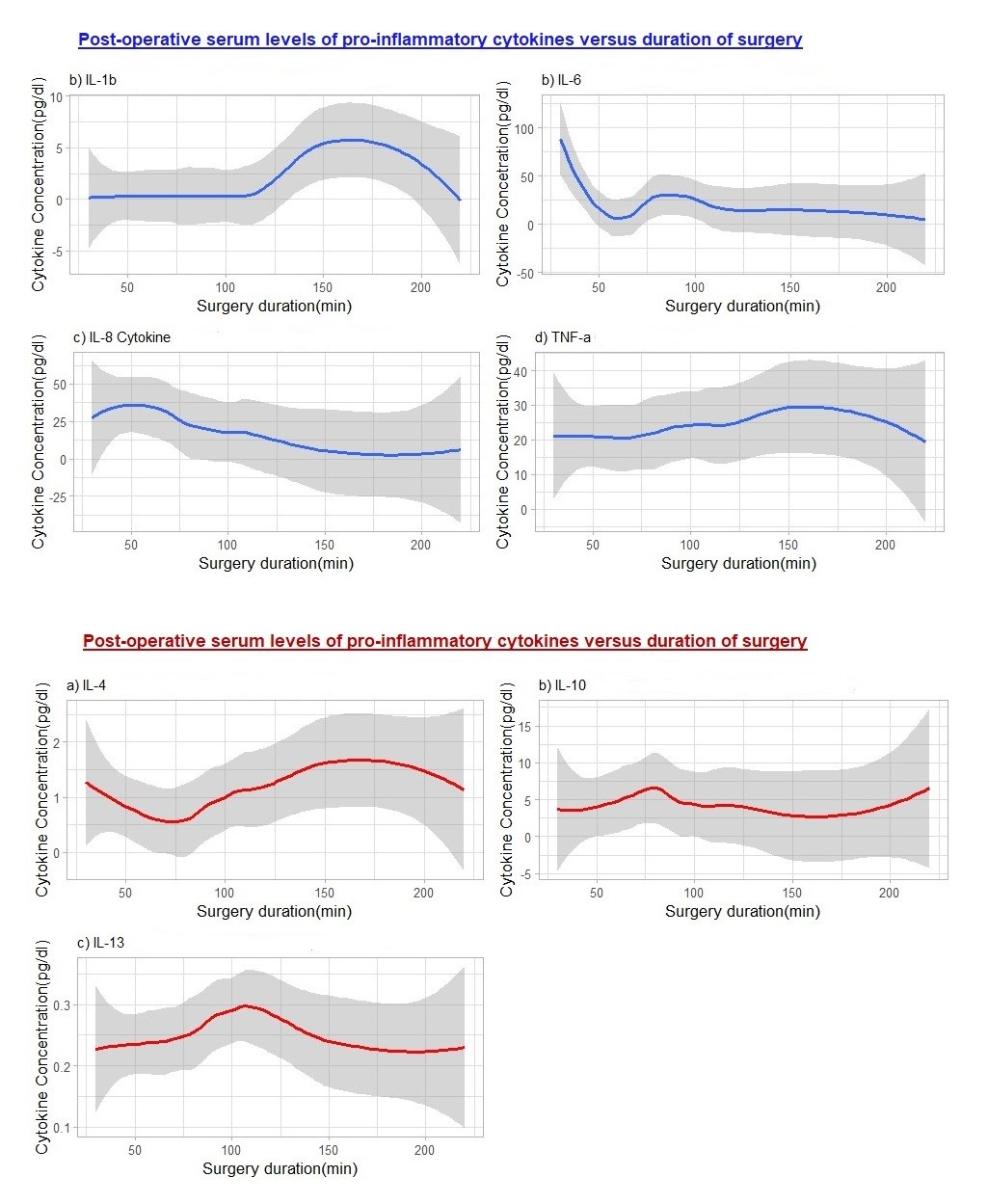

Supplement: Supplementary file 3 [file Image_3.JPEG]
